# An exploratory dosimetric and treatment-time analysis of tangent-arc and continuous semi-arc VMAT in deep inspiration breath-hold radiotherapy for stage I left-sided breast cancer

**DOI:** 10.1371/journal.pone.0348570

**Published:** 2026-05-08

**Authors:** Yucheng Li, Yinan Chen, Chaoqing Xu, Wenming Zhan, Kainan Shao, Yongshi Jia, Haibo Zhang, Lingyun Qiu, Jieni Ding, Weijun Chen

**Affiliations:** 1 Cancer Center, Department of Radiation Oncology, Zhejiang Provincial People’s Hospital (Affiliated People’s Hospital), Hangzhou Medical College, Hangzhou, Zhejiang, China; 2 School of Medical Technology, Yunnan Engineering Vocational College, Yunnan, China; 3 Center for Rehabilitation Medicine, Rehabilitation & Sports Medicine Research Institute of Zhejiang Province, Department of Rehabilitation Medicine, Zhejiang Provincial People’s Hospital (Affiliated People’s Hospital), Hangzhou Medical College, Hangzhou, Zhejiang, China; Northwestern University Feinberg School of Medicine, UNITED STATES OF AMERICA

## Abstract

**Background:**

The use of the deep inspiration breath-hold (DIBH) technique reduces cardiac and lung radiation exposure during left breast cancer radiotherapy. However, the optimal beam delivery technique and the effects of patient adaptation during DIBH remain incompletely understood.

**Objective:**

In this study, the dosimetric differences between continuous semi-arc and tangent-arc plans in stage I left-sided breast cancer patients using DIBH were compared, and the treatment session duration was descriptively analyzed to characterize treatment-time trends during routine DIBH delivery.

**Methods:**

Twenty patients treated at our hospital from 01/05/2022–31/05/2023 were retrospectively selected from the institutional database for exploratory dosimetric analysis. Two radiotherapy plans were created on the basis of each patient’s computed tomography (CT) images. Dosimetric parameters for the planning target volume (PTV) and organs at risk (OARs), and beam-on and total treatment times, were compared.

**Results:**

The conformity index (CI) for the PTV was significantly better with the continuous semi-arc plan (***P*** < 0.05), whereas the other PTV parameters did not significantly differ between the plans (***P*** > 0.05). The doses and beam-on time for all OARs (except the left ventricle) were significantly lower for the tangent-arc plan (***P*** < 0.05). Treatment time tended to stabilize across fractions, with a significant difference between the 15th and 16th sessions (***P*** < 0.05).

**Conclusion:**

With the tangent-arc plan, the beam-on time and radiation exposure to OARs were observed to be lower, while adequate PTV coverage was maintained in patients with stage I left-sided breast cancer using DIBH. Treatment times tended to stabilize with increasing treatment fractions. This observation suggests gradual patient adaptation during routine DIBH rather than a predefined training effect. Given the exploratory nature of these findings and the limited sample size from a single institution, these findings should be interpreted with caution and warrant further investigation in larger, multicenter studies.

## Introduction

Breast cancer is one of the most prevalent malignancies among women worldwide, and radiotherapy is a key adjuvant treatment [1–3]. In clinical practice, volumetric modulated arc therapy (VMAT) has been widely adopted; both continuous semi-arc and tangent-arc modes have been implemented and thus have drawn considerable research interest [4,5]. Studies have indicated that the incidence of coronary artery disease increases by 4–16% for every 1 Gy increase in the mean dose delivered to the heart [6–8]. Various strategies for minimizing radiation exposure to organs at risk (OARs) have been proposed, including technical improvements such as the use of image-guided techniques, the optimization of treatment plans, and the introduction of advanced irradiation techniques such as deep inspiration breath-hold (DIBH) technique, respiratory gating, and VMAT [9,10].

Research has highlighted the significant dosimetric advantages of VMAT in breast cancer radiotherapy. Hossain et al. [11] reported that VMAT not only ensures adequate coverage of the tumor target but also reduces the radiation dose to normal tissues. Li et al. [12] compared continuous semi-arc and tangent-arc plans in the treatment of left-sided breast cancer and reported that tangent-arc plans achieved greater reductions in radiation exposure to the OARs, such as the heart and lungs. In recent years, the integration of VMAT with the DIBH technique has been shown to markedly reduce radiation exposure to the heart and left anterior descending artery [13–15].

Although previous studies have investigated dosimetric differences between continuous semi-arc and tangent-arc techniques [12,16], in most studies, these techniques have been compared across independent patient cohorts using unpaired statistical analyses, thereby limiting the accuracy of their conclusions. Building upon our prior work [12], in the present study, we address this limitation by adopting a within-subject design in which each patient with stage I left-sided breast cancer received treatment plans based on both tangent-arc and semi-arc techniques. This paired approach minimizes interpatient variability and increases the statistical robustness of the dosimetric comparisons. Furthermore, while the DIBH technique has been shown to effectively reduce cardiac and pulmonary doses, its clinical implementation often faces challenges because of variations in patients’ breath-hold stability, particularly during the initial training sessions, Consequently, some patients require repeated training to achieve adequate compliance, which may reduce treatment efficiency. To date, few studies have systematically evaluated how the frequency of breath-hold training influences treatment stability and overall efficiency.

Therefore, we aimed to compare the dosimetric profiles of continuous semi-arc and tangent-arc plans in patients with stage I left-sided breast cancer patients using DIBH, and to explore how patient adaptation during routine DIBH sessions influences treatment efficiency without the implementation of a predefined breath-hold training protocol. These findings are intended to provide preliminary methodological insight into plan selection and to explore treatment time trends during routine DIBH implementation, rather than to directly guide clinical practice.

## Methods and materials

### Patient selection

In this retrospective study, 20 patients with stage I left-sided breast cancer treated at our hospital between 01/05/2022 and 31/05/2023 were selected using simple random sampling from the pool of eligible patients. The patients were between 33 and 58 years old, with an average age of 47.1 years. The inclusion criteria were as follows: clinical diagnosis of stage I breast cancer, no contraindications to radiotherapy, Karnofsky Performance Status (KPS) score greater than 70, age under 60 years, ability to fully understand and complete the DIBH process, and a breath-hold duration of more than 30 seconds. All patients successfully underwent computed tomography (CT) simulation and radiotherapy under DIBH conditions. DIBH was performed under surface-guided radiotherapy (SGRT) guidance using the Catalyst system to monitor and gate patient respiration. The exclusion criteria included a breath-hold duration of less than 30 seconds, communication difficulties, or underlying conditions that could affect the efficacy of radiotherapy. The requirement for informed consent was waived by the Ethics Committee of Zhejiang Provincial People’s Hospital, as this study involved the retrospective analysis of anonymized data. The waiver was explicitly approved by the ethics committee (Approval Number: QT2024249). Data analysis was conducted between 01/11/2024 and 30/11/2024 after ethics approval was obtained.

### CT simulation positioning and target contouring

All patients were positioned supine on a vacuum cushion with both arms raised for CT simulation (slice thickness, 5 mm). The clinical target volume (CTV) was expanded by 5 mm to generate the planning target volume (PTV), which was retracted 5 mm subcutaneously. PTV-boost was defined based on residual tumor or high-risk areas. Target and OAR contouring were performed by a senior physician following the guidelines of the Radiation Therapy Oncology Group (RTOG) and the International Commission on Radiological Units (ICRU) [17,18].

### Plan design and optimization of VMAT techniques

**Fig 1:**
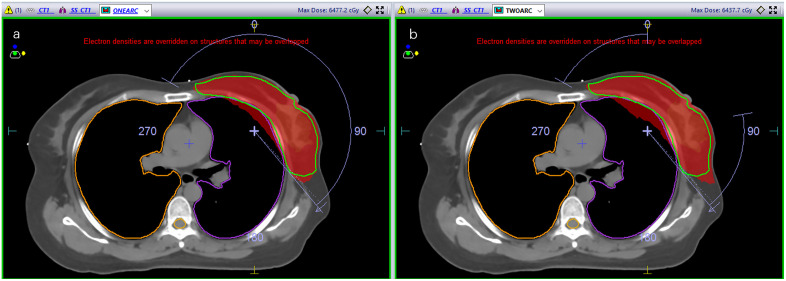
Schematic representation of two arc techniques used in radiation therapy: (a) Continuous semi-arc (left) and (b) Tangent-arc (right).

For each patient, two VMAT plans were generated based on their CT images: a continuous semi-arc plan and a tangent-arc plan. In the continuous semi-arc plan, a single arc was used, starting at 145° (±5°) and ending at 300° (±5°). In the tangent-arc plan, two arcs were employed: the first arc started at 145° (±5°) and ended at 85° (±5°), and the second arc started at 0° (±5°) and ended at 300° (±5°), as illustrated in Fig 1.

All plans were created by the same senior medical physicist using the Monaco V5.11 (Elekta AB, Stockholm, Sweden) treatment planning system. The fractionation scheme was 49.95 Gy in 27 fractions, with a simultaneous integrated boost (SIB) to the tumor bed up to 59.4 Gy in 27 fractions. Dose constraints for organs at risk (OARs) are detailed in Table 1 [19,20]. Dual arcs were applied for all plans.

**Table 1:** Dosimetric criteria for OARs.

OARs	Dosimetric Criteria
L-Lung	V_5_ < 55%
	V_20_ < 30%
	V_30_ < 20%
	D_mean_＜10 Gy
R-Lung	V_5_ < 20% D_mean_<2.5 Gy
Heart	^D^_mean_<8 Gy
L-ventricle	^D^_mean_<5 Gy
Spinal cord	D_max_ < 40 Gy

Except for the beam angles, all planning parameters were kept identical between the two plans, including OAR dose constraints, minimum subfield area (5 cm³), multileaf collimator (MLC) leaf spacing (0.5 cm), maximum number of subfields (200), and collimator angle (10°). The field size was determined according to the dimensions of the PTV. Optimization was performed using dose–volume histograms (DVHs) to ensure that doses to all OARs remained within predefined safety limits.

After plan creation, the radiation oncologist and physicist jointly reviewed each plan to confirm that all dose constraints for the PTV and OARs were met. For clinical delivery, all patients were treated using the tangent-arc plan, whereas continuous semi-arc plans were generated retrospectively for dosimetric comparison. The PTV prescription dose was normalized to 95%, such that the D95% encompassed the entire PTV. After plan approval, treatments were delivered with a 6-MV photon beam on an Infinity linear accelerator (Infinity6201; Elekta AB, Stockholm, Sweden).

### Dose evaluation

The primary parameters compared included the D_2_, D_98_, and D_mean_ values of the PTV (where D_x_ refers to the minimum dose received by x% of the volume of the corresponding target/organ, and D_mean_ indicates the mean dose received by the corresponding target/organ); V_5_, V_20_, V_30_, and D_mean_ for the left lung (where V_x_ represents the volume receiving at least an x Gy dose of radiation); V_5_ and D_mean_ for the right lung; D_mean_ for the heart and left ventricle; D_max_ (maximum dose) for the spinal cord; and the beam-on time, which was directly obtained from the optimization console of the Monaco treatment planning system upon completion of the treatment plan design. Additionally, the conformity index (CI) and homogeneity index (HI) of the PTV were calculated. The CI was calculated via the following formula:, where TV95 is the target volume receiving 95% of the prescription dose, TV is the target volume, and V95 is the volume receiving 95% of the prescription dose [21]. The HI was calculated as where D5% and D95% represent the doses received by 5% and 95% of the PTV, respectively [22].

### Workflow description and treatment time evaluation for DIBH

Prior to each treatment, patients were positioned on the treatment couch by a radiation therapist using the Catalyst surface-guided DIBH system to ensure accurate alignment. The system monitored patient motion in six dimensions, with setup criteria defined as translational deviations ≤5 mm and rotational deviations ≤3°. If these thresholds were exceeded, the therapist repositioned the patient until alignment met the criteria. Once positioning was confirmed, the therapist exited the treatment room.

**Fig 2:**
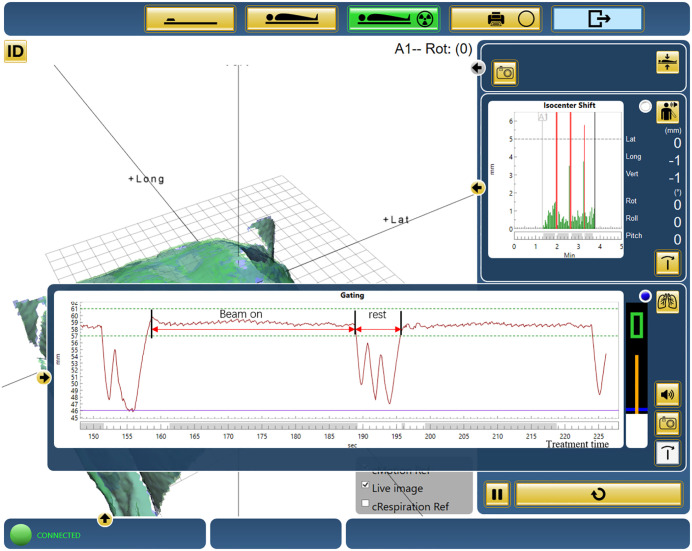
Treatment time recorded using Catalyst software for a single patient.

DIBH treatments were initiated via the “Response” gating interface within a predefined gating window, which controlled beam delivery on the linear accelerator (Fig 2). The Catalyst system provided real-time monitoring of respiratory motion and automated beam control.

Treatment time was defined as the total duration from the therapist’s instruction to “start treatment” until the completion of the entire plan. This included both beam-on time and patient breath-hold/rest intervals. Treatment times for all patients were recorded directly from the Catalyst system for subsequent analysis.

### Statistical analysis

Statistical analyses were performed using SPSS version 20.0 software (SPSS Inc., Chicago, IL, USA). The Kolmogorov–Smirnov (K–S) test was used to evaluate the normality of all the data. For variables that satisfied the assumptions of normality and homogeneity of variance, paired-sample t tests were used to compare parameters between different conditions (e.g., FB vs. DIBH) or between adjacent treatment sessions within the same patient cohort. The results are presented as the deviations. Since all comparisons were within-subject and limited in number, no correction for multiple testing was applied. Given the exploratory nature of this study, no formal correction for multiple testing was applied. The analyses were intended to identify preliminary patterns and effect directions to guide future research rather than to provide definitive statistical confirmation. ***P*** < 0.05 was considered to indicate statistical significance. The graphs were generated using GraphPad Prism 5.

## Results

With respect to the PTV, a significant difference between plans was observed only for the CI (***P*** < 0.05), while no statistically significant differences were found for the other parameters (***P*** > 0.05). With respect to the OARs, all the parameters, except those for the left ventricle, exhibited statistically significant differences between plans (***P*** < 0.05). The beam-on time for the tangent-arc plan was significantly shorter than that for the continuous semi-arc plan (***P*** < 0.05). Detailed parameter comparisons between the continuous semi-arc and tangent-arc plans are provided in Tables 2 and 3.

**Table 2:** Comparison of dosimetric parameters for OARs and PTV between continuous semi-arc and tangent-arc plans（‾χ ± s）.

parameters	continuous semi-arc	tangent-arc	*P*
PTV D_2%_ (Gy)	63.44 ± 6.65	62.26 ± 1.01	0.455
PTV D_98%_ (Gy)	47.77 ± 0.35	47.66 ± 0.58	0.627
PTV D_mean_ (Gy)	52.63 ± 0.74	52.81 ± 0.91	0.156
CI	0.80 ± 0.05	0.77 ± 0.05	0.007
HI	1.25 ± 0.03	1.26 ± 0.03	0.195
L-Lung V_5_ (%)	39.70 ± 4.10	37.80 ± 4.20	0.024
L-Lung V_20_ (%)	16.52 ± 2.70	17.30 ± 2.60	0.024
L-Lung V_30_ (%)	10.90 ± 2.20	12.10 ± 2.20	0.002
L-Lung D_mean_ (Gy)	9.52 ± 1.10	9.68 ± 1.03	0.301
R-Lung V_5_ (%)	6.46 ± 3.98	3.03 ± 3.00	<0.001
R-Lung D_mean_ (Gy)	2.29 ± 0.43	1.57 ± 0.46	<0.001
Heart D_mean_ (Gy)	4.40 ± 1.11	3.74 ± 1.14	<0.001
Left-Ventricle D_mean_ (Gy)	4.42 ± 1.36	4.16 ± 1.47	0.057
Spinal Cord D_max_ (Gy)	3.83 ± 2.67	1.69 ± 0.74	<0.001
Beam-on time (s)	94.85 ± 10.89	77.20 ± 11.80	<0.001

**Table 3:** Comparison of beam-on times between the continuous semi-arc and tangent-arc plans with different field angles.

Planning Technique	Arc Range (°)	Beam-On Time (s)
continuous semi-arc	145–325	67–112
tangent-arc（1st arc）	145–85	24–50
tangent-arc（2nd arc）	25–325	24–51

**Fig 3:**
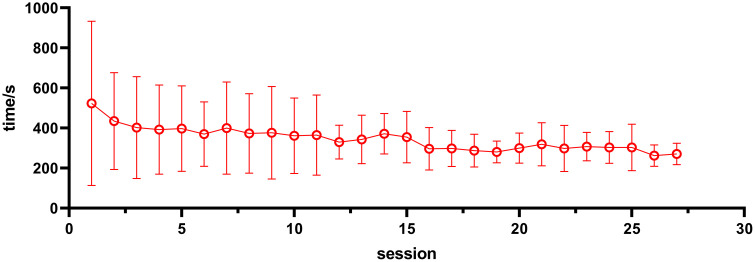
Trend of treatment time variation for all patients across each treatment session.

Statistical analysis of adjacent treatment times revealed notable fluctuations in the mean values across sessions, although no statistically significant differences were observed between most consecutive sessions. Overall trend analysis indicated that the mean value was highest during the first treatment session (522.50 s ± 410.41 s), followed by a gradual decrease over subsequent sessions, reaching the lowest value at the 26th session (262.00 s ± 53.49 s). Despite this trend, most pairwise comparisons between consecutive sessions did not reveal statistically significant differences. For example, there was no significant difference between the mean values of the 1st and 2nd sessions (***P*** = 0.252) or those of the 2nd and 3rd sessions (***P*** = 0.408). A statistically significant difference was observed between the 15^th^ (354.45 ± 128.22 s) and 16^th^ sessions (296.55 ± 106.08 s, P = 0.038). The absolute difference of 57.9 s corresponds to a small-to-moderate effect size (Cohen’s d ≈ 0.49), suggesting that while the change is statistically detectable, it is unlikely to represent a clinically meaningful improvement in workflow efficiency. In contrast, pairwise comparisons for the majority of the other sessions revealed no significant differences, as exemplified by the comparisons between the 3rd and 4th sessions ***(P*** = 0.883) and the 24th and 25th sessions (***P*** = 0.993). The comparison details are presented in Supplemental S1 Table. The trend in treatment time across treatment sessions for all patients is depicted in Fig 3.

## Discussion

As radiotherapy techniques have evolved, VMAT combined with DIBH has been widely adopted to reduce cardiac and pulmonary radiation exposure while maintaining adequate target coverage in left-sided breast cancer radiotherapy [23–25]. In this study, we aimed to evaluate the dosimetric differences between continuous semi-arc and tangent-arc radiotherapy plans in patients with stage I left-sided breast cancer who used DIBH and to analyze treatment time differences across sessions. Using the CT images from 20 patients, we developed both continuous semi-arc and tangent-arc radiotherapy plans and compared the dose distributions to the target volumes and OARs and recorded treatment time per session. The results indicated that compared with the continuous semi-arc plan, the tangent-arc plan not only reduced the beam-on time but also achieved superior dose control for most OARs while still ensuring adequate target coverage. Moreover, the treatment time tended to decrease after approximately the 15th session, suggesting a gradual adaptation to the DIBH technique during routine treatment delivery rather than a defined training effect. These findings are exploratory and hypothesis-generating, and further studies are needed to determine whether specific breath-hold training strategies or predefined adaptation periods can meaningfully influence treatment efficiency.

Pulmonary dose–volume parameters such as V20 and V5 are commonly used indicators of lung toxicity risk [26–28]. In this study, the mean left lung V20 remained below 20% for both planning techniques, suggesting acceptable pulmonary dose levels. Compared with the continuous semi-arc plan, the tangent-arc plan further reduced the lung V5, indicating a dosimetric advantage in limiting low-dose exposure. However, given the absence of pulmonary function data or clinical toxicity endpoints, the clinical implications of these differences should be interpreted cautiously.

Similarly, compared with the continuous semi-arc plan, the tangent-arc plan resulted in a statistically significant reduction in the mean heart dose. Although prior studies have demonstrated an association between incremental increases in mean heart dose and long-term cardiac risk, the modest absolute dose difference observed in this study cannot be directly translated into a measurable reduction in cardiac morbidity. This highlights the importance of distinguishing between statistical significance and clinical relevance when interpreting dosimetric findings. Building on our previous work evaluating the dosimetric advantages of tangent-arc and semi-arc techniques in breast radiotherapy [12], this study further advances the field by addressing key limitations of previous research, in which these techniques were often compared across independent patient cohorts using unpaired statistical analyses. In contrast, we adopted a within-subject design in which plans for each patient were developed using both techniques, enabling paired t test analysis, which reduced interpatient variability and increased the statistical robustness of the results. Because both planning techniques were generated within the same institutional workflow and evaluated using identical contour sets for each patient, the paired comparative design further minimizes the potential influence of inter-operator variability and primarily reflects relative technical differences between planning approaches. Moreover, we systematically examined the effect of breath-hold practice on treatment efficiency and patient compliance and reported that treatment times tended to stabilize around the 15th session. Together, these methodological approaches improve the reliability of the dosimetric comparison and provide exploratory insights into plan selection and treatment time adaptation during DIBH.

Breath-hold stability is essential for DIBH-based radiotherapy. In the tangent-arc plan, irradiation was divided into shorter arcs, enabling each arc to be completed within one to two breath-hold cycles and allowing patients to rest between arcs, thereby increasing efficiency without compromising accuracy. In contrast, the continuous semi-arc plan required multiple longer cycles, increasing fatigue and reducing reproducibility. These findings suggest that plan design may influence treatment efficiency during routine DIBH delivery, but the effects of a structured pretreatment breath-hold training protocol are poorly understood.

The mean treatment time was longest during the first treatment session (522.50 ± 410.41 s) (Supplemental S1 Table and Fig 3), likely reflecting patients’ initial unfamiliarity with the DIBH procedure. Treatment times gradually decreased and stabilized across subsequent sessions. Although a statistically significant reduction was observed between the 15^th^ and 16^th^ sessions, the absolute difference of 57.9 s corresponds to a small-to-moderate effect size (Cohen’s d ≈ 0.49) and is unlikely to represent a clinically meaningful improvement in workflow efficiency. No other consecutive sessions showed statistically significant differences (P > 0.05), and overall treatment times stabilized across subsequent sessions. These findings primarily reflect progressive patient familiarization and increased procedural consistency during routine DIBH delivery rather than definitive workflow optimization. Overall, the stabilization of treatment time across fractions should be interpreted as descriptive evidence of patient adaptation rather than the effect of a structured training intervention.

Several limitations of this study should be acknowledged. First, although patients were randomly selected, this randomization was based on retrospective case selection from an institutional database rather than prospective treatment allocation. Second, no predefined or structured breath-hold training protocol was implemented; thus, the observed stabilization of treatment time across fractions reflects gradual patient adaptation during routine DIBH rather than an interventional training effect. Third, inter-operator variability in target delineation and treatment planning was not quantitatively assessed. Although standardized institutional protocols were followed by experienced clinicians and physicists, residual variability may have influenced dose distribution, particularly for structures with complex anatomical boundaries. Such variability could contribute to small differences in dosimetric parameters and therefore the observed results should be interpreted as technique-related trends within a consistent institutional workflow rather than absolute effects independent of operator influence. Fourth, the mean maximum heart distance (MHD) observed in this cohort was greater than that typically reported in routine clinical practice, which may limit the generalizability of the dosimetric findings. Fifth, this study focused exclusively on dosimetric parameters and treatment efficiency, and did not assess clinical outcomes such as toxicity, cardiac function, or patient-reported measures; therefore, the dosimetric advantages cannot be directly translated into clinical benefit. In addition, multiple dosimetric and temporal endpoints were analyzed without formal correction for multiple testing, increasing the risk of type I error, this was considered appropriate given the exploratory and hypothesis-generating intent of the study. The results should therefore be interpreted as preliminary patterns rather than definitive statistical evidence. However, the observed effects demonstrated consistent directional trends across treatment fractions with effect magnitudes exceeding within-group variability, supporting the statistical stability of the findings despite the modest sample size. Finally, the relatively small sample size and single-center, retrospective design may further limit the statistical power and generalizability of the findings, warranting confirmation in larger prospective multicenter studies. Accordingly, given the exploratory design and hypothesis-generating intent of this study, the analyses were not intended to provide definitive statistical confirmation but rather to identify preliminary patterns and effect directions to inform future adequately powered investigations.

## Conclusion

Compared with the continuous semi-arc plan, the tangent-arc technique was associated with lower OARs doses and reduced beam-on times while maintaining adequate target coverage in patients with stage I left-sided breast cancer who used DIBH. Analysis of treatment time across fractions revealed a stabilization trend, reflecting gradual patient adaptation during routine treatment rather than a predefined training effect. As clinical outcomes such as toxicity, cardiac function, or patient-reported measures were not assessed, the translational relevance of these findings remains limited. Given the exploratory nature of this analysis and the absence of multiple testing correction, the results should be interpreted cautiously. Further prospective studies in larger, multicenter cohorts are warranted to confirm the robustness of these observations and to clarify the relationship between dosimetric advantages, treatment-time patterns, and clinical outcomes during DIBH radiotherapy.

## Supporting information

S1 Table Summary of Treatment Sessions with Statistical Comparisons. (RAR)
